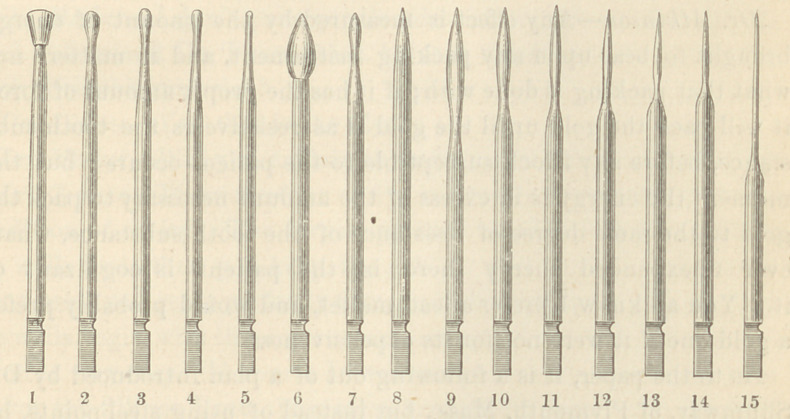# Central Association of Northern New Jersey

**Published:** 1884-02

**Authors:** 


### CENTRAL ASSOCIATION OF NORTHERN NEW JERSEY.

The regular monthly meeting of the Central Dental Association of Northern New Jersey, was held at the office of Dr. Sanger, Brick Church, N. J., on the evening of Thursday, November 22d, 1883.

The minutes of the previous meeting were read and approved.

*Dr. Timme*—read a paper upon "A New Method of Filling Teeth," and afterwards exhibited to the members the various instruments referred to in the paper.

Dr. Timme's paper was published in the January Number of the INDEPENDENT PRACTITIONER, and a cut of the instruments exhibited is given below.—*Editor.*

1 2 3 4 5 6 7 8 9 10 11 12 13 14 15

*Dr. Bodecker*—About a year ago, some gentlemen from Germany sent me the transactions of the local society at Frankfort-on-the-Main, in which Dr. Herbst explains the subject of Dr. Timme's paper. I have thought over this subject a great deal, as I believe it promises good results, but as I have not had the gold here, which is claimed to be a different preparation from that which we possess in this country, I have not experimented any. There were at that time two or three gentlemen in the meeting who spoke very highly of it, and who, I believe, have a very good standing in Europe. The instruments that are passed around here are nothing but smooth burnishers, and Dr. H. claims that by this method he is able to fill a cavity in half the time that is required in the ordinary way. There is, however, one mistake in Dr. Timme's paper, and that is this. He believes that ordinary cohesive gold cannot be manipulated well with smooth points. I have used them for at least six or seven years, and whenever I get new points in a condition that they do not work satisfactorily, I grind them off with sand-paper. The only difficulty I find with smooth points is that they become covered with gold very easily, and when they do, I just rub them off again with sand-paper, and then obtain the very best results I can possibly wish for.

*Dr. Osmun*—As Dr. Atkinson is the father of the mallet, I would like to hear from him upon this question of the mallet *vs.* burnishing gold in a cavity.

*Dr. Atkinson*—Any effect is measured by the amount of energy brought to bear upon any packing instrument, and it matters not what that packing is done with; if it has the proper amount of force it will pack the gold until the gold is as resistive as the tooth substance, before any shock susceptible to the patient occurs ; but the moment the energy is in excess of the amount necessary to pack the gold to the same degree of resistance of the tooth substance, whatever unexpended energy there is, the patient is cognizant of it. You all know I prefer a lead mallet, and would probably prefer a gold one if it were not for its expensiveness.

As to the paper, it is a following out of a plan introduced by Dr Shumway, of Plymouth, Mass., but instead of using steel points, he uses ivory points, and claims that he can fill a tooth with them in less time than he can with the mallet. I think our preferences are too personal to be of any considerable value if we were going to put it to vote; but if we comprehend the principle, and are willing to see which is best adapted to our own special organism, and understand all the laws of mechanics and chemistry involved in the filling of teeth, we need not be very strenuous about the method. The time was when I was a stickler for using the mallet always, and I think the world was benefited by that, because my enthusiasm in it gave me such bull-headed confidence in the perception of the truth, that I had cheek enough to sow it everywhere with such religious fervor that it burned itself into the minds of the dental profession.

I don't know that there is anything that is really desirable in the way of speech-making, that would not be tedious upon this subject. I hold that the best method of teaching that has ever been devised is that of questions and answers, when we have a certain number of minds engaged in a like pursuit, all honestly desiring the truth for itself (we usually want the truth for *our*selves), and when we really do want that, and have the humility to say that the truth is not mine, nor thine, but ours, and seek for it, then we can ask questions in such a manner as to call forth replies that will enable us to catch the principle involved in the questions much better than by long preaching.

*Dr. Meeker*—I don't quite get the idea in regard to this filling. Do I understand Dr. Timme that he uses the engine in filling the teeth with this material?

*Dr. Timme*—Yes, that is the idea.

*Dr. Meeker*—Will you explain to us the use of these instruments?

*Dr. Timme*—There are four or five instruments, according to the condition of cavity. The largest point is to press the gold against the walls first; then you keep the gold in position with an instrument until you get it perfectly firm, and after you get it solid against the walls begin with this instrument just as I described—it is No. 1. I find it very easy. Of course I have not very much experience with it, but the fillings I saw there were beautifully done. Dr. Herbst's brother did the most difficult work, filling the first and second bicuspids with ease, and I could not find anything to show the method would not be a good one.

*Dr. Osmun*—What is the peculiar method in the manufacture of this gold?

*Dr. Timme*—Dr. Herbst claims for it purity; nothing else.

*Dr. Osmun*—In thinking over this subject at short notice, it seems to me there must be one weak spot about it, and that is, getting it well under the undercut with those instruments. The filling must have some undercut, or shape of cavity of some kind, that it may withstand pressure, and if it is an ordinary cavity, that is one with nearly straight walls, there must be some weak spots. To my mind the gold cannot be thoroughly adapted to the walls of the cavity, and I fail to comprehend how he could get the gold solid with instruments that are run by the engine, with but light pressure, because if you run the engine rapidly, or even slowly, and put on much pressure, heat is generated, and the patient may make objections. He claims it is adapted to teeth of low organization. It seems to me that is the very kind of teeth it would be inappropriate to. Has Dr. Timme ever taken a tooth apart after it was filled, to see if it was a perfectly solid filling?

*Dr. Timme*—I did, and must say that the fillings seemed very solid, and the gold well impacted and fitted to all the inequalities of the cavity. It takes practice to do the work well, and I do not claim that I can do it perfectly. I would not trust myself to build up a tooth with it yet.

*Dr. Osmun*—How would you proceed with small proximal cavities?

*Dr. Timme*—Dr. Herbst does not use retaining pits, but makes slight undercuts if the cavity is not naturally retentive in shape. Then he proceeds upon the same general principle as in filling crown cavities.

*Dr. Osmun*—I should like to try it, but am afraid I am wedded to my sins.

*Dr. Meeker*—If you were building up an incisor, would you use those instruments?

*Dr. Timme*—No. I am afraid I cannot explain everything to you.

*The President*—Dr. Timme, in speaking of the instruments, will you call the numbers?

*Dr. Timme*—Numbers one, two, three and four are principally used for burnishing the surface, but they are also employed in laying the foundation of the filling, and securing the first layer of gold.

Number five is the principal instrument used, and there should be a number of sizes.

Number six may be used in impacting the gold into large angles of the cavity.

Numbers seven to fifteen are mainly used in approximal fillings. Number fifteen is simply a sewing needle cemented into a shaft that shall fit the engine. This is the most important of all the set, because it is mainly depended upon to see that there are no small unfilled pits or crevices.

The gold is used in the form of small cylinders, and unless it has been freshly made it may be annealed. The size of the point to be used will be indicated by the size and form of the cavity.

*Dr. Osmun*—If it is in order, I should like to ask Dr. Timme to give a clinic and invite the members of the Society to his office to witness it. I am interested in this subject, and if there is anything to be gotten out of it I would like to see it.

*The President*—Dr. Nicholi spoke very highly of this method. He was at first disgusted with it, but afterward he took it up again, and is now very much pleased with it. That is his statement.

*Dr. Meeker*—This paper of Dr. Timme's is very interesting. The only thing that I do not comprehend is how it is done. Of course if he will give a clinic I shall be pleased to attend it. I can very readily comprehend Dr. Shumway's method of using ivory points, because I have employed it frequently. I have a diamond disc, and sharpen up my instruments at nearly every filling. I never think of using a serrated plugger.

*Dr. Atkinson*—The statement that gold must be pure to be cohesive, is a mistake. Those who have experimented upon that have said that gold would be cohesive if it had even five per cent of silver in it, if it were only free from other impurities, and in that statement I have full confidence myself. I do not think cohesiveness always means pure gold; as high as ten per cent of silver may be in gold, and the foil or sheet be cohesive.

INCIDENTS OF OFFICE PRACTICE.

*The President*—A paper was read before this society by Dr. W. P. Richards, upon "Diseases of the Antrum and their treatment."

It was laid upon the table for discussion at this meeting. The subject is now in order.

Dr. Richards' paper was published in the December number of the INDEPENDENT PRACTITIONER.—*Editor.*

*Dr. Atkinson*—A drainage tube can only be useful when the natural drain is obstructed, and if you can reopen the natural passage it is the best drainage tube you can get. If the occlusion between the upper and middle, and lower and middle turbinated bones is complete, then you get dropsy of the antrum. If you do not succeed in opening the natural passages it may be advisable to use drainage tubes until such time as the parts return to a healthy condition, and in doing that you always want to make two openings, so as to put the syringe in one, and wash out of the other. We can always open between the roots of the teeth, say the second bicuspid and the first molar, and between the second and third molars, if they are all standing. Generally speaking, with proper care, an opening can be secured by the use of a plain spud put into the nostril, separating the occulting bones above and below.

When the bones are softened they must be removed in some way, and the best method is to remove them mechanically, when that is possible. There is a great deal of ambiguity in the minds of surgeons and dentists respecting the antrum. So far as disease thus located is concerned, its mechanical shape and locality is all that makes it different from any other mucous membrane, covering bones in the body, and you need not be at all afraid of it; all you have to do is to study well the anatomy of the parts. I have seen a great many openings made by surgeons directly into the chamber through the roof of the mouth, and always with poor results, and much discomfort to the patient during the healing process.

*Dr. Bodecker*—I have of late seen a large number of cases of disease of the antrum. Dr. Atkinson says it is not always necessary to use drainage tubes, but in some instances I have found them to be of the greatest value, and especially in those cases where by chronic catarrh the antral opening is partially obstructed, although there may not be an entire occlusion.

When the mucous membrane of the antrum is in a pathological condition there is usually more or less hypertrophy around that opening, and I usually make a drainage tube of platinum, to one end of which I solder a round shoulder, to prevent it from slipping into the antrum. If I have to make an opening, I generally make it very near the second bicuspid, or if that tooth is out, near the first molar, in the place where the second bicuspid came out ; then I fasten a clasp with a little movable projection around either the first molar or bicuspid. This projection answers two purposes: it prevents the entrance of food, and keeps it in its proper place without undue pressure. As a general rule, whenever I have used such drainage tubes, I have found that the disease abated in from three days to one week, especially if, before the tube is inserted, it is filled with very finely powdered iodoform, which by means of a syringe is blown into the antrum. But I have had one case which has given me a great deal of trouble. It was that of a young man about eighteen years of age, with a moderately good constitution. He came to me about two years ago to have his teeht filled. Upon examination I found that the root of the left upper first molar (the crown of which was broken off), would have to be removed, and consequently advised its extraction. A few days after the removal of the root, the patient told me that at times he observed great quantities of pus entering his mouth from the place where the root had been removed. I then examined it and found the antrum open and in a state of inflammation. I inserted a drainage tube and tried to syringe salt water through the antrum into the nose, but with no success, for I found that the naso-antral opening was completely obliterated. After further inquiry the young man told me that he had been suffering severely from hay fever for several years, and that during the whole winter he had been troubled with nasal catarrh, which I believe was the origin of the trouble in the antrum. After several weeks' treatment I found to my surprise that the communication of the antrum with the nose was not present yet, and as at that time there was considerable catarrh of the nasal cavity, I advised the patient to consult his physician about it. The nasal catarrh was soon cured, but the naso-antral opening remained closed as in the beginning of the treatment, which consisted of injections of a three per cent solution of boracic acid in water until the liquid run off clear, which was followed by either a solution of chloride of zinc, ten grains to one ounce of water, or a solution of nitrate of silver of the same strength. This treatment was continued every day for about two months, when I advised the patient to submit to the operation of having the naso-antral opening made by means of an instrument. The mother would not give her consent, in consequence of which the patient to-day is in precisely the same condition as in the beginning of the treatment. A few months ago I presented him at the clinic of the First District Dental Society of New York.

*Dr. Richards*—Do you always use metal tubes ?

*Dr. Bodecker*—If I intend them to remain for any great length of time I prefer platinum tubes. I never tried rubber.

*Dr. Richards*—Is there any irritation ?

*Dr. Bodecker*—I have never seen any irritation. Another good point about these tubes is that you can find out in a minute whether the nasal opening is obstructed, for if it is, when the syringe fits the drainage tube perfectly, no liquid is regurgitated from the antrum until the syringe is withdrawn from the tube. If on the other hand the opening is present, the liquid will enter the nasal cavity.

*Dr. Richards*—Do you not find a great advantage in the use of these drainage tubes ?

*Dr. Bodecker*—Yes. The more chronic the case is, the more necessary I regard the use of the drainage tube.

Under the head of " miscellaneous business " Dr. Watkins exhibited a diagram used in recording operations in the mouth, and in making examinations. Also a new form of tooth-brush, the handle of which is so shaped as to facilitate the cleaning of the posterior teeth.

Dr. Timme showed a new drop bottle.

Dr. Idel exhibited a switch-board, intended for use in deflecting the electric current from the motor to the electric mallet.

Upon motion the society adjourned for one month.

## Figures and Tables

**Figure f1:**